# Biologically synthesized silver nanoparticles as potent antibacterial effective against multidrug‐resistant *Pseudomonas aeruginosa*

**DOI:** 10.1111/lam.13759

**Published:** 2022-06-22

**Authors:** C. Campo‐Beleño, R.A. Villamizar‐Gallardo, L.E. López‐Jácome, E.E. González, S. Muñoz‐Carranza, B. Franco, R. Morales‐Espinosa, R. Coria‐Jimenez, R. Franco‐Cendejas, M. Hernández‐Durán, R. Lara‐Martínez, L.F. Jiménez‐García, A.M. Fernández‐Presas, R. García‐Contreras

**Affiliations:** ^1^ Departamento de Microbiología y Parasitología, Facultad de Medicina Universidad Nacional Autónoma de México Mexico City Mexico; ^2^ Departamento de Medicina, Facultad de Salud Universidad de Pamplona Cúcuta Norte de Santander Colombia; ^3^ Infectious Diseases Division Instituto Nacional de Rehabilitación Luis Guillermo Ibarra Ibarra Mexico City Mexico; ^4^ Centro de Ciencia y Tecnología Nanoescalar, “nanCiTec” Bogotá Colombia; ^5^ División de Ciencias Naturales y Exactas, Departamento de Biología Universidad de Guanajuato Guanajuato Mexico; ^6^ Laboratorio de Bacteriología Experimental Instituto Nacional de Pediatría Mexico City Mexico; ^7^ Departamento de Biología Celular, Facultad de Ciencias UNAM, Universidad Nacional Autónoma de México Mexico City Mexico

**Keywords:** activity, antimicrobials, mechanisms of action, pseudomonads, resistance

## Abstract

*Pseudomonas aeruginosa* is one of the most worrisome infectious bacteria due to its intrinsic and acquired resistance against several antibiotics and the recalcitrance of its infections; hence, the development of novel antimicrobials effective against multidrug‐resistant *P. aeruginosa* is mandatory. In this work, silver nanoparticles obtained by green synthesis using a leaf extract and fungi were tested against a battery of clinical strains from cystic fibrosis, pneumonia and burnt patients, some of them with multidrug resistance. Both nanoparticles showed a potent antibacterial effect, causing severe damage to the cell wall, membrane and DNA, and inducing the production of reactive oxygen species. Moreover, the nanoparticles derived from fungi showed synergistic antibacterial effects with the antibiotics meropenem and levofloxacin for some clinical strains and both kinds of nanoparticles were nontoxic for larvae of the moth *Galleria mellonella*, encouraging further research for their implementation in the treatment of *P. aeruginosa* infections.

## Graphical abstract

**Significance and Impact of the Study:** The continuous rise of antibiotic resistance in bacteria such as *Pseudomonas aeruginosa* is a serious threat to human health, hence it is mandatory to identify novel ways to combat its infections. In this work, silver nanoparticles were synthesized in a green way using leaves and fugal extracts and its antibacterial effects were tested against clinical strains. The nanoparticles killed the strains due severe cellular damage affecting the membranes, the DNA and generating reactive oxygen species, in addition they were nontoxic and displayed synergy with some antibiotics; therefore, being a promising alternative to treat MDR *P. aeruginosa* infections.

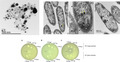

## Introduction

Current levels of antibiotic resistance in bacteria are alarming. Projections indicate that if there is no improvement in the treatment of infections caused by multidrug‐resistant bacteria (MDR), in 2050, there will be 10 million deaths per year due to these infections worldwide. Therefore, the development of new effective antibacterial drugs is required, particularly those against MDR strains of *Pseudomonas aeruginosa* (de Kraker *et al*. 2016; López‐Jácome *et al*. 2019).

*Pseudomonas aeruginosa*, is an opportunistic bacterium in patients with burns, cystic fibrosis and immunosuppressed individuals. The epidemiological importance is based on its remarkable ability to develop resistance to multiple antibiotics by diverse mechanisms such as efflux pumps, mutations in antibiotic targets, loss of porins, expression of antibiotic‐inactivating enzymes (broad‐spectrum β‐lactamases, metallo‐β‐lactamases), alteration of penicillin‐binding proteins, DNA‐gyrase mutations or due to the acquisition of resistance genes by horizontal genetic transfer (Peleg and Hooper 2010).

Consequently, in this sense, nanomaterials are promising new antimicrobial alternatives. Among them, silver nanoparticles (AgNPs) have been gaining interest due to their important properties such as chemical stability, catalytic activity, surface plasmon resonance and high conductivity (Dawadi *et al*. 2021). Nevertheless, the chemical methods for nanoparticle synthesis are reducing and stabilizing agents used, which are sometimes toxic and polluting, causing damage to the environment and human health, limiting their applications (Li *et al*. 2011). Therefore, developing reliable, nontoxic and environmentally friendly methods to synthesize nanomaterials is important to expand its biomedical applications (Zhang *et al*. 2016).

For this reason, nanoparticle biosynthesized using bacteria, fungi or plant extracts are preferred rather than traditional and less environmentally friendly methods (Arole and Munde 2014). And recently, other eco‐friendly methods for their synthesis, such as the use of electrochemical devices, have been developed (Pompilio *et al*. 2018).

The bactericidal effect of silver nanoparticles has been proposed to be linked to the direct contact of nanoparticles with the bacterial cell wall, followed by penetration into the cytoplasm. Direct contact of silver nanoparticles with large surface areas in a bacterial cell wall could induce membrane damage, lead and cell content release, and cell death (Barros *et al*. 2018).

Furthermore, silver nanoparticles can interact with the respiratory chain, generating reactive oxygen species (ROS), such as radicals, hydrogen peroxide (H_2_O_2_), hydroxyl (OH‐) and superoxide (O_2_‐) that induce oxidative stress and damage to proteins and nucleic acids. The nanoparticles also penetrate the cytoplasm and interact with proteins and DNA, causing cell death. ROS generation is responsible for bacterial death because it facilitates lipid peroxidation and inhibits ATP production and DNA replication. Similarly, silver nanoparticles can also release silver ions, which increase cell damage (Li *et al*. 2010). Silver ions released by AgNPs interact with phosphorous in DNA and with some proteins, resulting in inhibition of enzymatic activities (García‐Contreras *et al*. 2011). The antimicrobial action of Ag^+^ ions is closely related to their interaction with thiol (sulfhydryl) groups. Thus, Ag^+^ ions can react with the ‐SH groups of enzymes and proteins attached to the cell wall, interfering with the respiratory chain of bacteria and breaking the bacterial cell wall. As a result, DNA loses the ability to replicate, and the proteins essential for ATP production are disabled (Hsueh *et al*. 2015).

The antibacterial activity of silver nanoparticles is also related to the cell structure morphology of many types of bacteria. Gram‐negative bacteria are generally more sensitive to Ag^+^ invasion than Gram‐positive bacteria due to the difference in their cell wall structures; Gram‐positive bacteria have a very thick cell wall that contains several layers of peptidoglycan, which acts as a barrier to the penetration of Ag^+^ ions into the cytoplasm. In contrast, Gram‐negative bacteria only have a single layer of peptidoglycan, and due to this characteristic, Ag^+^ ions can easily damage the cell wall (Khalandi *et al*. 2017). In this context, metallic nanoparticles are considered promising agents, that produce a significant level of ROS, with the ability to overcome the microbial antioxidant defence system and, consequently, cause cellular damage (Ahmad *et al*. 2017). Recent studies have demonstrated the ability of silver nanoparticles to kill *P. aeruginosa* and other pathogenic bacteria such as *Burkholderia cepacia* and *Staphylococcus aureus* and to decrease biofilm formation (Salomoni *et al*. 2017; Pompilio *et al*. 2018; Espinosa *et al*. 2020; Huang *et al*. 2022). This research aimed to study the antibacterial properties of silver nanoparticles synthesized by two distinct pathways in multi‐resistant strains of *P. aeruginosa*, strains isolated from patients with cystic fibrosis, skin burns, and pneumonia with resistance against several antibiotics.

## Results and discussion

### Nanoparticle synthesis and characterization

Silver nanoparticles were synthesized for 4 h. After this time, a change in coloration was appreciated. The most accepted criterion to validate the synthesis of silver nanoparticles with silver ions, fungi, bacteria or plant extracts is the change in coloration (AbdelRahim *et al*. 2017).

The characterization of the nanoparticles was carried out by UV–Vis spectroscopy in the range of 300–700 nm. As expected, an absorption band in the UV–Vis spectral region (380–430 nm) was observed due to the silver nanoparticles' surface plasmon resonance (AbdelRahim *et al*. 2017). The peaks at 380 and 430 nm of the fungal‐synthesized nanoparticles (F‐NP) and leave‐synthesized nanoparticles (L‐NP) indicate they have a spherical shape (Amendola *et al*. 2010). The presence of a 400 nm peak corresponds to silver nanoparticles less than 5 nm in diameter. In contrast, an absorption peak of 420 nm indicates that the predominant size of the silver nanoparticles is around 20 nm.

Transmission electron microscopy (TEM) analysis was performed to determine additional characteristics of silver nanoparticles. This microscopy is a frequently used technique for characterizing nanomaterials to obtain quantitative particle size, size distribution and morphology measurements. The size of nanoparticles was from 5·34 to 70 nm, and the average size was 26·06 nm. The size distribution indicated that almost 50% of the silver nanoparticles were in the 20–30 nm size range and were approximately spherical (Fig. S1).

The most accepted mechanism for the biological formation of silver nanoparticles involves the enzymatic reduction by nitrate reductases. The micro‐organism or plant extract provides these enzymes, or the extract is used and requires NADH/NADPH as coenzymes. These enzymes catalyse the formation of silver nanoparticles only when the reaction is carried out under suitable conditions, depending on pH, temperature, reaction time, stirring speed, the substrate used and the light exposure during synthesis (Singh *et al*. 2013).

### Antibiotic resistance profiles of the used strains

All the strains included in the study were resistant to ceftazidime and had intermediate breakpoints to colistin; two‐thirds were resistant to amikacin, gentamicin and piperacillin/tazobactam, and half of them were resistant to the quinolones (ciprofloxacin and levofloxacin), and one‐third were resistant to meropenem (Table S1). Based on current definitions (Magiorakos *et al*. 2012) H039 and H278 are extensively drug‐resistant, RME 124 and INP‐62 are multidrug‐resistant.

### Minimal inhibitory concentration of nanoparticles and synergism with antibiotics

The minimal inhibitory concentration (MIC) of the NP was tested by the microdilution method, finding that MICs for F‐NP (between 4 and 128 μg ml^−1^) were similar to those for L‐NP (between 8 and >128 μg ml^−1^) (Table S1).

No significant changes for meropenem MIC in combination with nanoparticles were found; only the strain H039 had a reduction of MICs when F‐NP was added. However, for levofloxacin 3/6 (50%) NP had a significant reduction (at least three double dilutions) being the best combination. Nevertheless, a significant decrease in MICs values was observed only for F‐NP. Neither F‐NP nor L‐NP showed synergism with ciprofloxacin and colistin and no change in any antibiotic MIC was observed with L‐NP. All assays were performed by duplicate (Table S1).

### Exposure to the silver nanoparticles promotes severe bacterial damage

**Figure 1:**
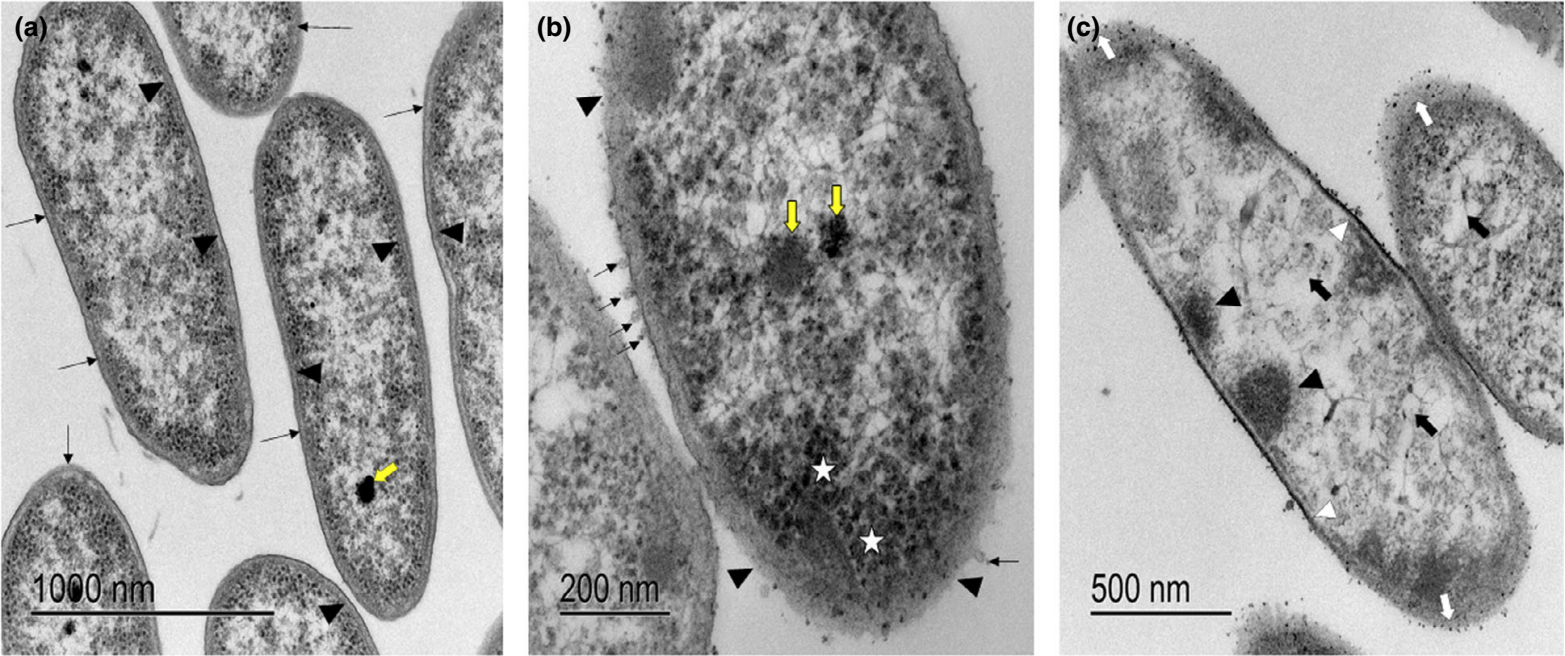
Micrographs of *Pseudomonas aeruginosa* (strain PA14), incubated with (a) LB culture medium (control), an intact cell wall (arrows) and well‐defined membranes (arrowhead) are observed, the cytoplasm electron density is homogeneous and DNA electron density is increased (yellow arrow). (b) Silver nanoparticles biologically synthesized by *Aspergillus flavus* 100% of bacteria exhibit extensive damage with breaks in the cell wall (arrowheads). Note blebbing in the cell wall (arrows), and ribosomes accumulation is also observed (white star), a diminished electron density in bacteria DNA (yellow arrows) when compared to untreated bacteria). (c) The nanoparticles are distributed along the entire cell wall, predominating in the anterior and posterior end of the bacteria, (white arrows) likewise, loss of the integrity of the membranes is observed (white arrowhead), accumulation of chromatin‐like attached to the plasma membrane (black arrowhead) is observed, and a decrease in the cytoplasm electron density due to the leakage of intracellular components (black arrow). [Colour figure can be viewed at wileyonlinelibrary.com]

The ultrastructural study of *P. aeruginosa* incubated with silver nanoparticles showed severe damage produced in the different structures of the bacterium. The results obtained from the incubation of *P. aeruginosa* in the absence of nanoparticles showed that the cell wall and the plasma membrane are well defined and intact. Furthermore, the cytoplasm's electron density and the bacterium's DNA were homogeneous, as shown in Fig. 1a. In contrast, bacteria treated with silver nanoparticles exhibited considerable damage, showing an accumulation of nanoparticles on the cell wall and at the bacterium's inner and outer poles. Likewise, the cell wall is scalloped and interrupted, the plasma membrane has interruptions, and in the cytoplasm, a granular pattern observed probably corresponds to bacterial ribosomes. Furthermore, clusters close to the plasma membrane are observed, probably chromatin and the electron density of the cytoplasm is decreased, with loss of cellular material, which is evidenced by the presence of clear areas as shown in (Fig. 1 panel c).

**Figure 2:**
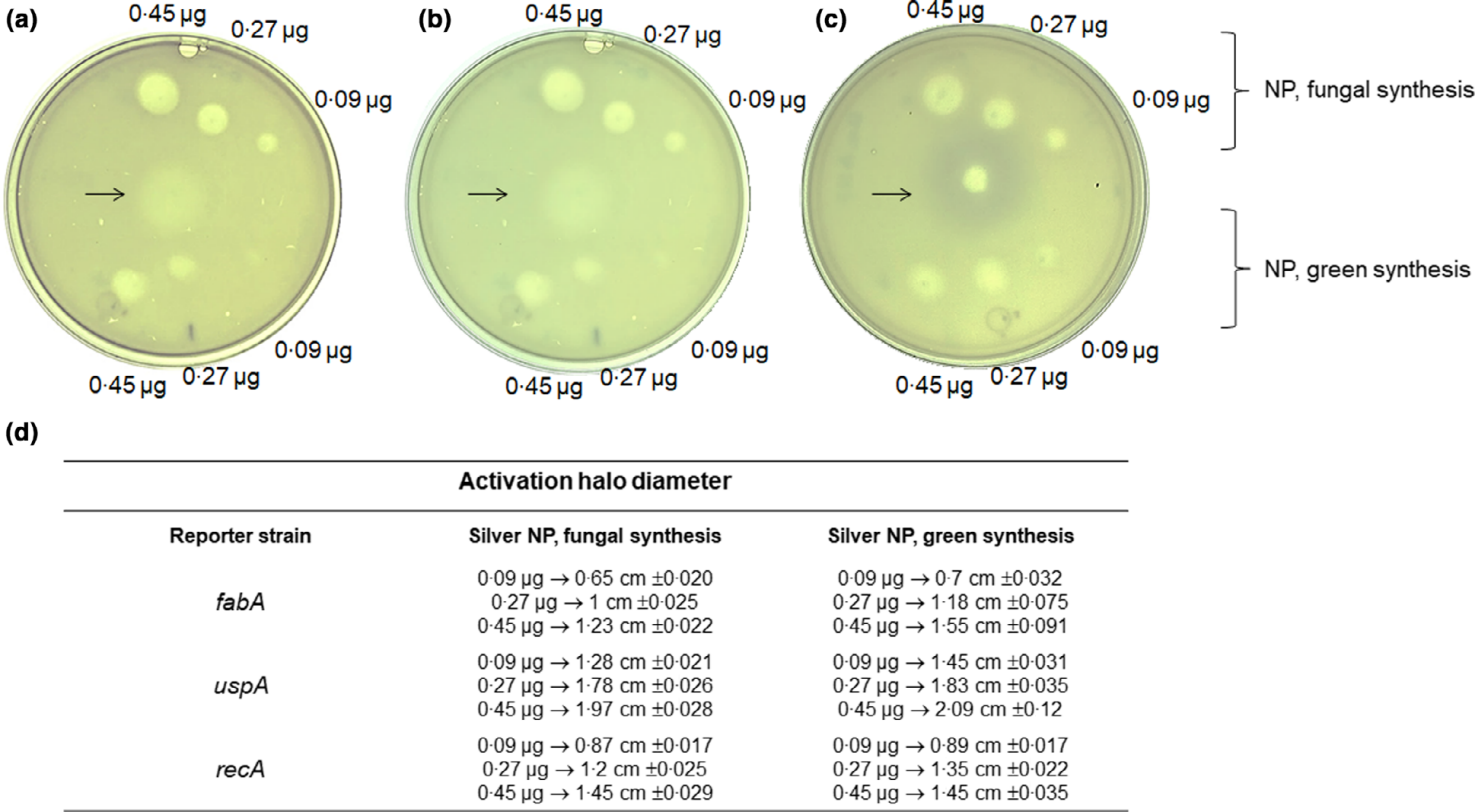
Reporter strain analysis for NP‐induced cell damage. In panel a, the effect of NP on the activation of the cell membrane was assessed with *fabA* reporter strain. In panel b, general NP‐induced cell stress was evaluated with the *uspA* reporter strain. In panel c, the NP‐induced DNA damage was assessed with the *recA* reporter strain. On each spot, different concentrations of NPs were deposited. In panel d, the diameter of each activation halo is indicated. Experiments were done in triplicates. The black arrow indicates the position where positive controls were placed. For *fabA* and *uspA*, SDS at 0·2% was used and for *recA* mitomycin C (0·1 μg μl^−1^) was used. [Colour figure can be viewed at wileyonlinelibrary.com]

The damage produced by the nanoparticles to the bacteria was also evidenced using biosensors designed to evaluate general stress, DNA damage and membrane disruption. The activation of the biosensors is recorded by forming a blue‐purple halo surrounding the growth inhibition zone, where the NP concentration is high. Still, with the diffusion on the surface, the remaining living cells with lower damage can activate the reporter. Results revealed both L‐NP and F‐NP induced cell death and general damage to both types of NP. In the case of DNA damage and membrane disruption, both NPs showed cell death and activation of the reporter, showing a greater for L‐NP (Fig. 2). In all instances, the bioreporter strains showed that NPs damaged the cell membrane and, once inside, damaged all components. This assay also shows that the NPs generated may produce ions that render activation at low concentrations of the NPs, which is found further from the site of placing the NPs.

### ROS generation

The evaluation of the oxidative state of the fluorescent probe dihydroethidium, revealed that both L‐NP and F‐NP induced the production of high concentrations of ROS in the two strains tested PA14 and RME 124, reaching similar levels to those induced by menadione at 25 mmol l^−1^ (Fig. S2). This agrees with the observed cell damage in TEM and with the biosensors since ROS induces lipid peroxidation and hydroxide radicals damage DNA generating double‐strand breaks and activating *recA*.

### Proposed killing action mechanism of silver nanoparticles

Remarkably, all strains tested were susceptible to LNP and FNP independently of their MDR nature. Based on the TEM micrographs, we can predict that the bactericidal effect starts from the direct contact of the silver nanoparticles with the cell wall and membrane; the disruption of these structures causes the release of cytoplasmic material and finally, cell death, as described previously in other studies (Barros *et al*. 2018). Likewise, silver nanoparticles can interact with the respiratory chain once they penetrate the bacteria's cytoplasm, thus promoting ROS generation that induces oxidative stress, denaturation or damage of proteins and nucleic acids (García‐Contreras *et al*. 2011). This was confirmed by the two reporter strains that show membrane and DNA damage (*fabA* and *recA* respectively), along with cell death in the place of direct contact with the NPs. Our results agree with the findings described by Baskaran and coworkers in 2016 using silver nanoparticles synthesized by the utilization of a cell‐free protein of *Rhizopus oryzae* extract (Ramalingam *et al*. 2016). And with the proposed action mechanisms of silver nanoparticles against *P. aeruginosa*: membrane disruption and ROS generation are elucidated through proteomic and computational approaches (Yan *et al*. 2018).

Another possible antibacterial mechanism of NPs is many sulphur‐containing proteins on the surface of the bacterial cell. Therefore, silver ions can interact with sulphur‐containing proteins inside or outside the cell membrane, which affects the viability of the bacterial cell. Silver ions act by replacing other metal ions necessary for the cell, such as Ca^2+^ or Zn^2+^. Parallel to this, silver ions may also interact with phosphorous residues in DNA, causing inactivation of the DNA replication, which was demonstrated in this work by showing DNA damage using a reporter strain. Also, silver ions can react with sulphydryl groups of metabolic enzymes of the electron transport chain, causing its inactivation (‘Vasil'kov *et al*. 2018).

### Silver nanoparticles are nontoxic for *Galleria mellonella*

Finally, the effect of both L‐NP and F‐NP was evaluated in *Galleria mellonella* larvae, showing that injecting 1 mg of NP is not toxic to the larvae since survival remained at 100% after 5 days (data not shown).

The lack of toxicity of the NP towards *G. mellonella*, encourages further research, such as the evaluation of their toxicity cytotoxicity and genotoxicity in mammal cell lines, as was previously done for commercial silver nanoparticles with antibacterial effect against *P. aeruginosa*, finding that they had no cytotoxic effects against cell lines (Salomoni *et al*. 2017), and its toxicity in mice, if the results confirm that the NP are safe, then its possible future application in the clinic should be pursued. For such a goal, the systemic administration of the particles seems difficult to implement, especially regarding the possible accumulation and damage of tissues and organs, but by either coating of catheters and prosthetic devices or their topical application for the treatment of skin infections and the prevention of disease in burnt patients may be the future for antibacterial NP that are cost‐effective for synthesis. In agreement with this proposal in a recent work, a novel biodegradable cryogel based on gelatine and silver nanoparticles was formulated and demonstrated good antibacterial and antibiofilm properties in vitro towards *P. aeruginosa* and *Staphylococcus aureus* and remarkably promoted wound healing and angiogenesis burn wounds infected with *P. aeruginosa* (Huang *et al*. 2022).

## Materials and methods

### Strains and culture conditions

The *Aspergillus flavus* strain, used for the biological synthesis of nanoparticles, was obtained from the Nanosost laboratory, located at the University of Pamplona, Colombia (Villamizar‐Gallardo *et al*. 2017).

*Pseudomonas aeruginosa* strains were obtained from the Instituto Nacional de Pediatría, Instituto Nacional de Rehabilitación Luis Guillermo Ibarra Ibarra and from Dr. Rosario Morales Espinosa from Faculty of Medicine, UNAM. The strains were isolated from patients with cystic fibrosis (strains INP‐62, INP‐64), burns (H278, H039) and pneumonia (RME 27 and RME 124).

Bacterial strains were cultured in LB broth (LB at 37°C, with 200 rev min^−1^ shaking for 24 h). The stock solution was prepared from this culture was adjusted to a concentration of 0·5 McFarland scale (1·5 × 10^8^ CFU/ml).

### Biological synthesis of silver nanoparticles with *Aspergillus flavus*

A stock solution of *A. flavus* was inoculated into a flask containing 150 ml of potato dextrose broth. Then, it was incubated at 25°C/100 rev min^−1^/8 days to obtain fungal biomass. After incubation, fungal biomass was filtrated using Whatman paper number 1 and kindly washed with distilled water to remove excess growth media. Cell biomass was collected and transferred to another flask containing 100 ml of sterile water to induce fungal stress and, therefore, the production of secondary metabolites such as reducing enzymes. The solution obtained was filtered again through a Whatman No. 1 filter and exposed to a solution of silver nitrate (AgNO_3_) (Sigma‐Aldrich, St. Louis, MO), 1 mmol l^−1^ pH 6·5 in v:v ratio. Then was kept at room temperature until the colour changed, indicating the biological synthesis of silver nanoparticles. The process was carried out by triplicate (Villamizar‐Gallardo 2016). These nanoparticles were denominated F‐NP.

### Green synthesis of silver nanoparticles with *Citrus latifolia tan*

The silver nanoparticles were synthesized using 5 mmol l^−1^ silver nitrate (AgNO_3_) (Sigma‐Aldrich) as the precursor of the metal salt, ascorbic acid and citric acid as reducing and stabilizing agents obtained from the extract of Tahiti lime (*Citrus latifolia tan*). The extract was prepared by filtering the fruit juice with a Whatman filter, heated at boiling temperature for 5 min, and centrifuged at 7000 ***g*** for 15 min to remove impurities. A percentage of citric acid equal to 4·8 ± 0·4% was determined by titration with 0·095 N sodium hydroxide and 1% phenolphthalein, while 0·4 ± 0·1% of the ascorbic acid concentration was determined by titration with iodine. A 1 : 3 (vol : vol) ratio of silver salt solution and the extract was allowed to react for 5 h at 25°C to obtain pristine nanoparticles (Gonzáles *et al*. 2015). These nanoparticles were denominated L‐NP.

### Characterization of silver nanoparticles

The silver nanoparticles were analysed by UV–vis spectrophotometry to observe the absorbance peak generated in a wavelength range between 350 and 700 nm. The microscopic characterization of nanoparticles was carried out with a transmission electron microscope (TEM), to obtain a detailed result on their morphology and size (Joshi *et al*. 2008).

### Evaluation of antibacterial effects *in vitro*

#### Determination of the minimal inhibitory concentration of the nanoparticles

The MIC was determined by the broth microdilution method using a 96‐well plate according to CLSI M07 recommendations using Mueller Hinton broth adjusted with cations. Nanoparticles were tested from 0·125 to 128 μg ml^−1^ for all strains. *P. aeruginosa* ATCC 27853, which is referred such strain used in quality control in the M100 document, was included (CLSI 2020). These experiments were done by duplicate, and MIC was defined as the concentration where bacterial growth is not visible.

#### Strains susceptibility and synergy between nanoparticles and antibiotics

For each strain included the MIC for amikacin, gentamicin, ceftazidime, ciprofloxacin, levofloxacin, meropenem, colistin and piperacillin/tazobactam were performed. Antibiotics concentration tested ranged from 0·062 to 64 μg ml^−1^ for all except for piperacillin/tazobactam (128/4–0·125/4 μg ml^−1^). Breakpoints and methodology were followed according to CLSI recommendations (CLSI 2020). Once the MIC was obtained for F‐NP and L‐NP, antibiotics MICs were estimated, and then synergism between NP and antibiotics was determined. For both cases, the concentration used was 128 μg ml^−1^. Synergisms were evaluated only for ceftazidime, levofloxacin, and meropenem in the same concentration. A final concentration of nanoparticles was synthesized with fungus supernatants of 0·5 mg ml^−1^ and for those obtained by green synthesis of 6·5 mg ml^−1^. We considered such synergistic activity when the MIC was at least two serial dilutions down compared to the antibiotic alone.

#### Effect of silver nanoparticles against *Pseudomonas aeruginosa* through TEM

*Pseudomonas aeruginosa* samples were processed before and after exposure to silver nanoparticles and treated as follows: a bacterial suspension of 1.5 × 10^8^ CFU per ml was incubated in LB culture medium for 24 h at 37°C (control) another sample was incubated with F‐NP. The bacteria were washed three times with fresh PBS, pH 5·2 at 4°C, resuspended in 0·15 mol l^−1^ cacodylate, and fixed with Karnovskýs solution for 1 h at room temperature. Subsequently, they were transferred to 0·1 mol l^−1^ cacodylate buffer, fixed in 1% osmium tetroxide, then dehydrated in ethanol and propylene oxide (1 h) and included in poly/bed 812/DMP30. The sections were photographed using a Jeol JEM 1200 EXII transmission electron microscope (Fernández‐Presas *et al*. 2018).

#### Generation of reactive oxygen species (ROS)

Reactive oxygen species generation (superoxide and H_2_O_2_) upon the treatment with AgNPs were determined fluorometrically by following the oxidation of 5 mmol l^−1^ of dihydroethidium (DHE; Sigma‐Aldrich) (excitation 480 nm/emission 530 nm) in 48 well plates, every 2 min using a Perkin Elmer Victor Nivo plate reader. DMSO, the diluent used for DHE, was used as a negative control, and 25 mmol l^−1^ of menadione was used as a positive control. Bacteria with DHE but not additional compounds were used to determine the rate of basal ROS generation. The ROS generation rate was determined from the slope of the plot of fluorescence vs time and the effect of NP was calculated by subtraction the rate of ROS generation without NP from the rate with NP. The slope of the negative control was subtracted from the slopes of the bacteria treated with NP.

#### Evaluation of cell and DNA damage using biosensors

The bacterial cell, and DNA damage induced by the nanoparticles were evaluated using biosensors containing the promoter regions of the genes *uspA, recA* and *fabA*, fused to the blue chromo‐protein AmilCP, which construction was described in Mora‐Garduño et al. (2022). To determine general stress, DNA damage and membrane damage respectively, a biosensor reporter strain was used. The biosensor strains were plated on top of agar media containing 100 μg ml^−1^ ampicillin. NP was spotted on top of the plate, placing 0·09, 0·27 and 0·45 total μg put on each spot. As positive controls, SDS and Mitomycin C were used to activate membrane damage and stress response (*fabA* and *uspA* reporter) and DNA damage (*recA* reporter). The nature of the reporter is to obtain a qualitative signal of activation. Plates were incubated 20 h after placing the NPs and activation was recorded using a digital camera.

#### Toxicity and evaluation of antibacterial effects *in vivo* using *Galleria mellonella*

Larvae of the insect *Galleria mellonella* were used for the *in vivo* toxicity test of the two types of NPs. The assay was carried out in groups of five larvae. A volume of 10 μl was taken from L‐NP and F‐NP at concentrations of 6·5 and 0·5 mg ml^−1^, respectively, and the larvae were injected with a u‐100 ultra‐fine 6 mm insulin syringe (BD, Franklin Lakes, NJ). As a negative control, the same volume was injected with 0·9% sterile saline solution, and as a positive control, 10^8^ bacterial cells suspension prepared with the strain PA14. Larvae were incubated at 37°C without access to food or water, and their survival was determined daily for 5 days after injection modified from Rossoni *et al*. (2019). Animals were manipulated according to the institutional animal care facility regulations.

## Authors contribution

R G‐C, AM F‐P, R V, LE L‐J, EE G, R C‐J, R F‐C, R M‐E and BF designed the study, C CB, EE G, S M‐C, M H‐D, LE L‐J, R L‐M, LF J‐G, AM F‐P and R G‐C performed the experiments, C C‐B and R G‐C wrote the manuscript with input of all other coauthors.

## Conflict of interest

We declare no conflict of interest.

## Supporting information


**Figure S1**. Micrographs of the obtained F‐NP, scale bar 200 nm.
**Figure S2**. Reactive oxygen species (ROS) generation relative to menadione 25 mmol l^−1^, both L‐NP and F‐NP were added to the reference strain PA14 and the clinical strain RME 124 at concentrations of 65 and 50 μg ml^−1^ respectively. ROS generation was recorded by following the oxidation of 5 mmol l^−1^ of dihydroethidium.

**Table S1**. Minimal inhibitory concentrations of antibiotics and nanoparticles and their synergisms.

## Data Availability

All crude data is available upon request.
