# Supplementary figures and images for: Biologically synthesized silver nanoparticles as potent antibacterial effective against multidrug‐resistant Pseudomonas aeruginosa
Source: Lett Appl Microbiol. 2022 Jun 22;75(3):680–8. doi: 10.1111/lam.13759 (PMC9543579; doi:10.1111/lam.13759)

## Slide 1
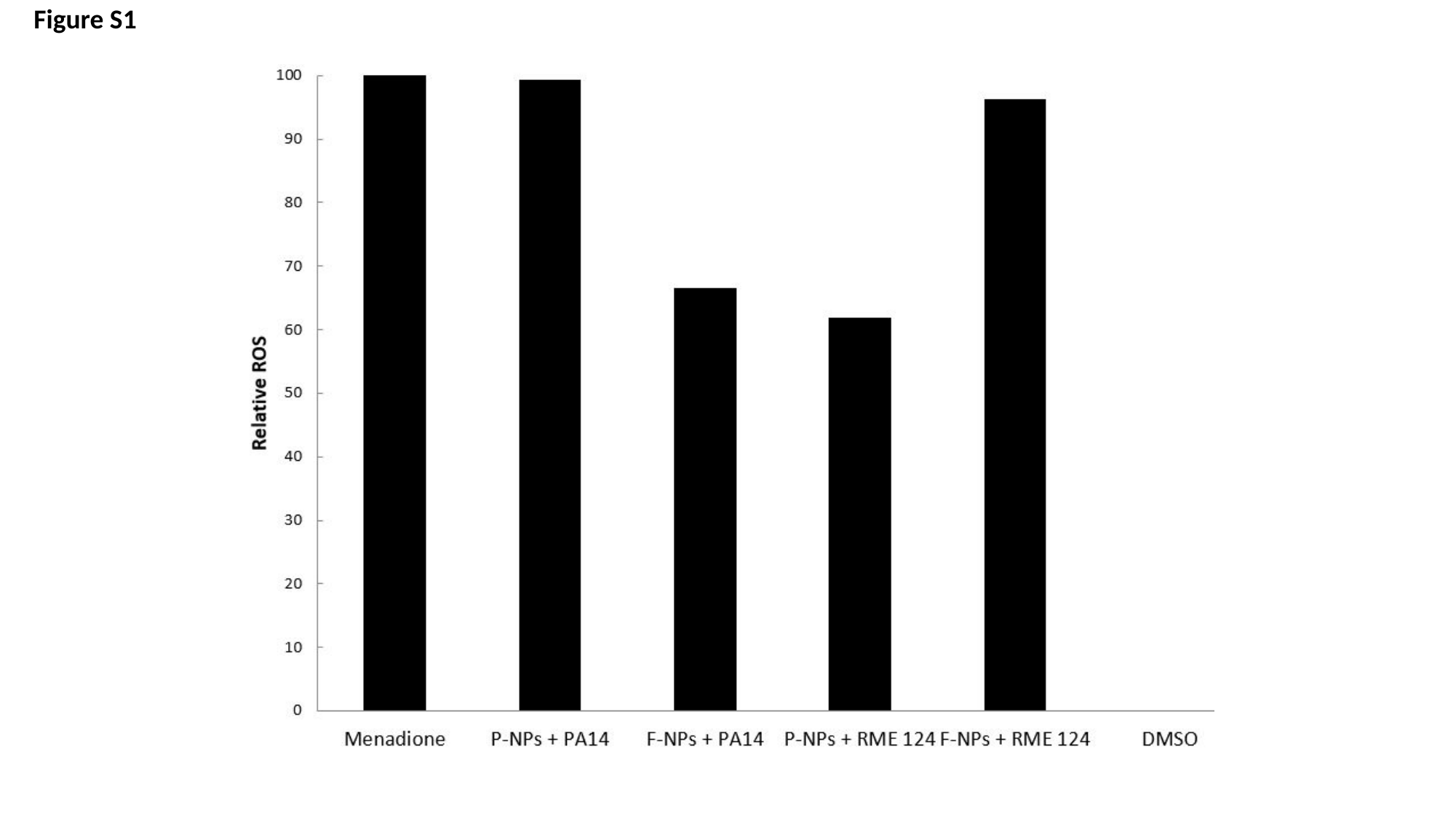

Figure S1

## Slide 2
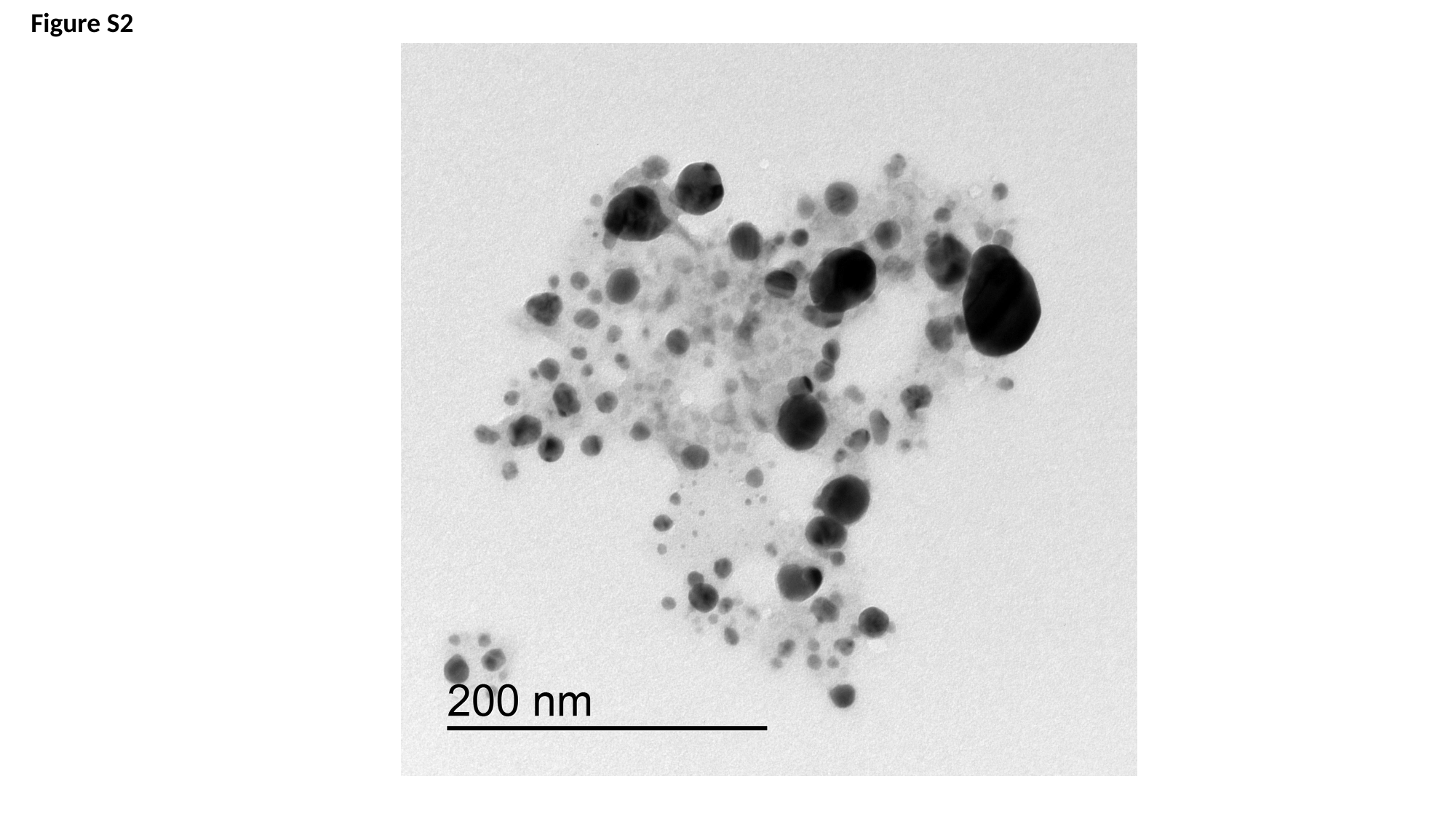

Figure S2

Supplement: Supplementary file 1 — Figure S1. Micrographs of the obtained F‐NP, scale bar 200 nm. Figure S2. Reactive oxygen species (ROS) generation relative to menadione 25 mmol l−1, both L‐NP and F‐NP were added to the reference strain PA14 and the clinical strain RME 124 at concentrations of 65 and 50 μg ml−1 respectively. ROS generation was recorded by following the oxidation of 5 mmol l−1 of dihydroethidium. [file LAM-75-680-s002.pptx]
